# Cerebos Salt

**Published:** 1907-06-15

**Authors:** 


### Practical Points.

### CEREBOS SALT.

Patients often ask one what cerebos salt is, and whether it is as good for them as common salt. We think that the one and only point in which cerebos is superior to ordinary salt is one which appeals to the mistress of a house and to her tablemaid. It is quite dry, and remains so even in wet weather; in consequence of this it is easy to make it tidy in the salt cellar, it does not cake, and it does not so readily blacken the silver and make this difficult to clean.

In regard to that for which salt is taken—its taste, its saltness—cerebos is quite inferior to ordinary salt; it is much less salt to the taste; it takes much more of it to give the flavour of saltness; cerebos is not likely to replace common salt in the kitchen as a flavouring agent.

In regard to its solubility one has only to put a teaspoonful of it into a tumbler of water and watch it. Part of the cerebos dissolves readily enough, but a considerable other part remains as a white deposit, undissolved even after hours. Ordinary salt consists almost entirely of sodium chloride, with a small and variable admixture of other compounds, particularly magnesium chloride. It is the deliquescent magnesium chloride in common salt which, absorbing moisture from the air in damp weather, leads to caking afterwards, and all the little troubles there are in dealing with ordinary salt in the salt cellars. Cerebos is simply ordinary salt to which a certain proportion of phosphates have been added. These phosphates convert the deliquescent magnesium chloride into the dry magnesium phosphate, thereby obviating some of the difficulties of the tablemaid; but this admixture diminishes the "saltness" of the whole; and it is the insoluble phosphates of lime and magnesia which remain at the bottom of a tumbler of water to which cerebos has been added.

Patients ask if the cerebos can do them harm, and the answer is clearly that it will not; at the same time the deficiency in saltness and the deficiency in solubility are two disadvantages which tend much to counterbalance the advantage of the dryness of the powder.

